# Dr. Thomas M. Winterbottom

**Published:** 1859-09

**Authors:** 


DR. THOMAS M. WINTERBOTTOM died on the 8th of July, from peritonitis and intestinal obstruction, in the ninety-fourth year of his age. Early in his life he held the position of Physician to the Colony of Sierra Leone, and afterwards wrote a work entitled "An Account of the Native Africans in the Neighborhood of Sierra Leone." He was not an extensive writer, but contributed some articles to the Edinburgh Medical and Surgical Journal. His fortune, amounting to over one hundred thousand dollars, has been left to trustees, for the establishment of a marine school for the education of seafaring men in the higher branches of navigation.

